# Mastectomy with immediate breast reconstruction: Results of a mono-centric 4-years cohort

**DOI:** 10.1016/j.amsu.2020.12.033

**Published:** 2020-12-31

**Authors:** Olivia Quilichini, Julien Barrou, Marie Bannier, Sandrine Rua, Aurore Van Troy, Laura Sabiani, Eric Lambaudie, Monique Cohen, Gilles Houvenaeghel

**Affiliations:** a Department of Surgery, Paoli Calmettes Institute & CRCM & Aix Marseille Univ, 232 Bd Ste Marguerite, Marseille, France; b Department of Surgery, Paoli Calmettes Institute, 232 Bd Ste Marguerite, Marseille, France

**Keywords:** breast, Reconstruction, Mastectomy, Complication, Robotic

## Abstract

**Introduction:**

Oncological safety, quality of life and cosmetic outcomes seems to be similar between breast conserving surgery (BCS) and mastectomy with immediate breast reconstruction (IBR). We report our experience of IBR for consecutive mastectomies realized in a recent period of four years in order to determined immediate surgical results according to type of mastectomy and type of reconstruction, as mains objectives.

**Methods:**

All mastectomies with IBR during years 2016–2019 were included. A retrospective analysis with prospective data collection was performed.

**Results:**

We analyzed 748 IBR: 353 nipple-sparing mastectomies (NSM), 391 skin-sparing mastectomies (SSM) and 4 standard mastectomies, 551 with definitive implant or expanders and 196 with latissimus dorsi-flap (LDF). More NSM were performed during the 2 last years and more LDF were performed for high BMI, high breast cup-size, neo-adjuvant chemotherapy and radiotherapy and local recurrence. We realized 111 robotic NSM and 125 robotic LDF. Longer duration of surgery was significantly associated with the robotic procedures.

The overall complications crude rate was 31.4% with 9.9% of re-operations and 5.8% of implant loss. Grade 2–3 complications were significantly associated with smoking. Breast complications occurred in 32.9% of mastectomies with principally skin or nipple-areola-complex suffering or necrosis, hematomas and infections. A predictive score was determined to evaluate risk of complications before surgery.

**Conclusion:**

Mastectomy with IBR seems to be a safe technique with an acceptable complication rate which is increased by tobacco use, high breast cup-size and IBR-type.

## Highlights

• Mastectomy with immediate breast to determined immediate surgical results.
• More Nipple Sparing Mastectomy performed during the 2 last years.
• More Latissimus dorsi-flap performed for high BMI, high breast cup-size, after radiotherapy.

## 1 Introduction

Breast-conserving surgery (BCS) for breast cancer (BC) has increase since numerous years and recently with development of oncoplasty and re-operation only for non in-sano resection. However total mastectomies for BC were still required in 12%–30% of patients [[1], [2], [3]]. Mastectomies can be required for extended ductal carcinoma in-situ (DCIS), multifocal disease, large BC according to breast size without indication of neo-adjuvant chemotherapy (NAC), prophylactic mastectomies, ipsilateral BC local recurrence (ILBCLR), non in-sano initial resection and patient's wishes. Secondary mastectomy for non in-sano BCS was realized in 40.8%–58.4% [4].

In France, immediate breast reconstruction (IBR) rate was lower than others European countries but has been increasing for several years [5]. Until now, IBR indications were: extended or multifocal DCIS, ILBCLR and prophylactic mastectomy [6] but was discussed for BC requiring adjuvant chemotherapy or radiotherapy [7].

In a recent French prospective study, satisfaction with the cosmetic outcome strongly influenced quality of life and an unsatisfactory outcome after IBR was still considered a better condition than simple mastectomy [8]. In the US, variable rates of breast reconstruction were reported, depending a great deal on where patients lived, what kind of health insurance they had and her race/ethnicity [9].

Reconstruction with implant or latissimus dorsi-flap (LDF) is usually proposed according to patient's wishes, previous treatment, patient's morphology, breast cup-size and ptosis. Moreover, since a few years' robotic mastectomy and or robotic LDF-IBR has been proposed [[10], [11], [12], [13], [14], [15], [16], [17]].

Several publications proved the benefit of risk reducing nipple-sparing-mastectomy (NSM) in high risk patients [[18], [19], [20]], with a 90% reducing in BC development [18]. NSM studies reported better esthetic results than skin-sparing-mastectomy (SSM) and better quality of life [21,22]. Otherwise, quality of life and cosmetic outcomes seems to be similar between BCS and IBR [23]. NSM with IBR is considered as a valid procedure for prophylactic mastectomy and an acceptable option for BC [[24], [25], [26], [27]]. Consequently, the demand of NSM by patients and the propositions of NSM by surgeons increased [28,29]. However few prospective studies were reported to evaluate complication rates and oncological outcomes of NSM [29].

The main technics of IBR are definitive implant or tissue expanders and LDF. In a French study, IBR was performed in 404 patients (67.9%), with implants in 46.5% and LDF in 46.9% [8]. Complications rates ranged between 5 and 61% are difficult to compare between studies due to great disparities of IBR types, type of complications recorded, indications of mastectomy and time of survey.

We report our experience of IBR for consecutive mastectomies realized in a recent period of four years in order to determined immediate surgical results according to type of mastectomy and type of reconstruction, as mains objectives.

## 2 Methods

### 2.1 Cohort study design

Among all consecutive mastectomies performed during years 2016–2019, we select patients with IBR from breast institutional database. A retrospective analysis with prospective data collection was performed in order to determined immediate surgical results according to type of mastectomy and reconstruction. Institutional committee approved this study (ClinicalTrial.gov n° NCT03461172 Database of Data Collection (BDD-G3S), Paoli Calmettes Institute, Marseille, France).

The work has been reported in line with the STROCSS [30].

### 2.2 Patients and outcomes

We analyzed patient's characteristics such as age, body mass index (BMI), breast cup-size, ASA status (American Society of Anesthesiology score), diabetes and tobacco use. Tumor characteristics or prophylactic treatment, previous treatment received (neo-adjuvant chemotherapy, radiotherapy), surgical procedures of mastectomy and IBR, complications have also been listed.

Data were collected regarding patient and tumor characteristics, treatment received and years of treatment, surgical procedures of mastectomy and IBR, complications during post-operative 90-days.

Analyses were performed separately for all complications, breast complications, LDF complications and for endoscopic surgical procedures. Technics of endoscopic NSM and robotic LDF were reported previously [[15], [16], [17]]. Complication rate was analyzed with Clavien-Dindo grading [31]: Grade 3 corresponded to any complication which required re-operation and Grade 4 corresponded to severe general infection. Grade 1 or 2 complications corresponded to infection or dehiscence or hematoma or bleeding or skin necrosis, without re-operation.

The duration of surgery was recorded from skin incision to the end of skin suture. The number of post-operative hospitalization days was reported from day of surgery to discharge. Interval-time between surgery and adjuvant chemotherapy (AC) or post-mastectomy radiotherapy (PMRT) were analyzed.

### 2.3 Procedures

Several techniques of IBR have been used for both NSM and SSM: sub-pectoral implant, tissue expanders or LDF; traditional open technique or robotic technic have been listed. The surgeon according to his habits chose incision. Patients underwent mastectomy with NAC conservation when distance between tumor and NAC was at least 2 cm on the preoperative imagery and a retro-mammary biopsy was performed.

### 2.4 Statistics

Quantitative criteria were analyzed with median, mean, CI95% and range. Comparisons were determined using Chi2-test for qualitative criteria and *t*-test for quantitative criteria. Factors significantly associated with criteria analyzed were determined by binary logistic regression adjusted for all significant variables determined by univariate analysis. Using Odds Ratio derived from logistic regression, we calculated a score for prediction of complications. Performance of this score was analyzed with calculation of area under the ROC curve (AUC). Statistical significance was set as *p* ≤ 0.05. Analyses were performed with SPSS version 16.0 (SPSS Inc., Chicago, Illinois).

## 3 Results

### 3.1 Population

We performed 1982 mastectomies: 1234 without IBR (60 bilateral: 4.9%) and 748 with IBR (37.7%) (134 bilateral: 17.9%). Characteristics of patients, surgery and treatments were reported in Table 1. Seven surgeons performed 728 mastectomies with IBR (97.3%) and 4 others surgeons performed 20 mastectomies with IBR.

**Table 1:** Characteristics of patients, surgery and treatments according type of mastectomy.

744 mastectomies		NSM		SSM		Chi2
excluding 4 standard mastectomies		Nb	%	Nb	%	p
Age	≤ median 49	201	56.9	180	46.0	0.002
	>49 years	152	43.1	211	54.0	
Indication	Primary	214	60.6	332	84.9	<0.0001
	Local recurrence	39	11.0	46	11.8	
	Prophylactic	100	28.3	13	3.3	
Bilateral	No	258	73.1	352	90.0	<0.0001
	Yes	95	26.9	39	10.0	
Years	2016	72	20.4	87	22.3	<0.0001
	2017	55	15.6	116	29.7	
	2018	118	33.4	104	26.6	
	2019	108	30.6	84	21.5	
Radiotherapy	No	248	70.3	251	64.2	0.019
	PMRT	51	14.4	55	14.1	
	Previous RTH	40	11.3	47	12.0	
	NAC + N-RTH	14	4.0	38	9.7	
Neo-adjuvant	No	311	88.1	331	84.7	0.104
chemotherapy	Yes	42	11.9	60	15.3	
Tobacco	No	270	76.5	320	81.8	0.044
	Yes	83	23.5	71	18.2	
Diabetes	No	350	99.2	386	98.7	0.420
	Yes	3	0.8	5	1.3	
ASA status	1	156	44.3	181	46.3	0.857
	2	189	53.7	203	51.9	
	3	7	2.0	7	1.8	
Breast cup size	A-B	222	62.9	191	48.8	0.001
	C	88	24.9	128	32.7	
	> C	43	12.2	72	18.4	
Histology	DCIS	54	15.3	99	25.3	<0.0001
	Ductal	150	42.5	221	56.5	
	Lobular	47	13.3	55	14.1	
	Others	3	0.8	2	0.5	
	No cancer	99	28.0	14	3.6	
Axillary surgery	No	184	52.1	132	33.8	<0.0001
	SLNB	140	39.7	206	52.7	
	ALND	29	8.2	53	13.6	
Implant	Definitive	261	95.6	198	71.2	<0.0001
	Expender	12	4.4	80	28.8	
LDF	no autologous	30	38.0	23	20.4	0.006
	autologous	49	62.0	90	79.6	
LDF±implant	without implant	56		88		
	with implant	23		25		
Robotic NSM	No	242	68.6	391	100	<0.0001
or endoscopic	Yes	111	31.4	0	0	
Robotic LDF	No	4	5.1	63	55.8	<0.0001
	Yes	75	94.9	50	44.2	

*Legend*: PMRT: post mastectomy radiotherapy, RTH: radiotherapy, NAC: neo-adjuvant chemotherapy, N-RTH: neo-adjuvant radiotherapy, DCIS: ductal carcinoma in-situ, SLNB: sentinel lymph node biopsy, ALND: axillary lymph node dissection, LDF: latissimus dorsi-flap, NSM: nipple sparing mastectomy. No autologous LDF: LDF without fat around muscle. Autologous LDF: LDF with fat around muscle.

### 3.2 Indications of mastectomies and neo-adjuvant treatments

Mastectomies were realized for 548 primitive BC (73.3%), 87 ILBCLR (11.6%) and 113 prophylactic mastectomies (15.1%) with bilateral mastectomies in 60 cases (10.9%), 7 cases (8%) and 67 (59.3%) respectively.

Neo-adjuvant chemotherapy (NAC) was performed before 104 mastectomies (13.9%) including 52 with NAC and neo-adjuvant radiotherapy (NAC-R) and including 2 standard mastectomies. Previous radiotherapy was realized in 89 mastectomies (11.9%) and in 52 mastectomies with NAC-R (7.0%).

### 3.3 Type of mastectomies

We realized 353 NSM (47.2%), 391 SSM (52.3%) and 4 standard mastectomies. NSM rate increased during the last 2 years: 56.5% (236/418) versus 38.5% (127/330) (p < 0.0001).

In univariate analysis, NSM versus SSM, was significantly associated with median age, breast cup-size, indication and histology, years of treatment, bilateral mastectomy and tobacco (Table 1). In multivariate analysis, we reported more SSM for breast cup-size C (OR: 1.642, CI95% 1.134–2.379, p = 0.009) or > C (OR: 1.780, CI95% 1.109–2.856, p = 0.017), primary BC (OR: 4.570, CI95% 1.345–15.52, p = 0.015) and less SSM during the two last years (OR: 0.617, CI95% 0.389–0.977, p = 0.039 and OR: 0.511, CI95% 0.318–0.820, p = 0.005) and for patients with tobacco use (OR: 0.673, CI95% 0.455–0.996, p = 0.048).

Incisions for SSM were central, around nipple areolar-complex (NACx) or elliptic. Incisions for 353 NSM were: 200 periphery breast incisions (56.7%: 147 axillar or external fold and 53 inferior fold), 88 with areolar or areolar and radial incisions (24.9%), 54 radial incisions (15.3%) and 11 with inversed T incision (3.1%).

### 3.4 Type of reconstruction

IBR were performed in 551 mastectomies with implants (459 definitive implants and 92 expanders), in 196 with LDF (including 48 with concomitant definitive implant) and in 1 with exclusive secondary lipofilling. IBR were performed with implant in 72.1% of primary BC (Table 2).

**Table 2:** Characteristics of patients, surgery and treatments according type of immediate breast reconstruction.

1 patient excluded: lipofilling only		IBR with implant		IBR with LDF		Chi2
		Nb	%	Nb	%	p
Age	≤ median 49	303	55.0	79	40.3	<0.0001
	>49 years	248	45.0	117	59.7	
Indication	Primary	395	71.7	153	78.1	<0.0001
	Local recurrence	44	8.0	42	21.4	
	Prophylactic	112	20.3	1	0.5	
Bilateral	No	417	75.7	196	100	<0.0001
	Yes	134	24.3	0		
Years	2016	119	21.6	40	20.4	<0.0001
	2017	112	20.3	59	30.1	
	2018	151	27.4	71	36.2	
	2019	169	30.7	26	13.3	
Radiotherapy	No	427	77.5	73	37.2	<0.0001
	PMRT	78	14.2	29	14.8	
	Previous RTH	46	8.3	42	21.4	
	NAC + N-RTH	0	0	52	26.5	
Neo-adjuvant	No	515	93.5	128	65.3	<0.0001
chemotherapy	Yes	36	6.5	68	34.7	
Tobacco	No	153 p	80.6	148	75.5	0.082
	Yes	107	19.4	48	24.5	
Diabetes	No	549	99.6	190	96.9	0.005
	Yes	2	0.4	6	3.1	
ASA status	1	268	48.7	69	35.2	0.005
	2	273	49.6	123	62.8	
	3	9	1.6	4	2.0	
Breast cup size	A-B	324	58.8	88	44.9	0.003
	C	150	27.2	67	34.2	
	> C	77	14.0	41	20.9	
Histology	DCIS	121	22.0	32	16.3	<0.0001
	Ductal	252	45.7	122	62.2	
	Lobular	66	12.0	35	17.9	
	Others	1	0.2	4	2.0	
	No cancer	111	20.1	3	1.5	
Axillary surgery	No	229	41.6	89	45.4	<0.0001
	SLNB	292	53.0	54	27.6	
	ALND	30	5.4	53	27.0	
Robotic NSM	No	497	90.2	139	70.9	<0.0001
or endoscopic	Yes	54	9.8	57	29.1	

*Legend*: PMRT: post mastectomy radiotherapy, RTH: radiotherapy, NAC: neo-adjuvant chemotherapy, N-RTH: neo-adjuvant radiotherapy, DCIS: ductal carcinoma in-situ, SLNB: sentinel lymph node biopsy, ALND: axillary lymph node dissection, LDF: latissimus dorsi-flap, NSM: nipple sparing mastectomy.

Median size of implants was 290 cc (mean 291, CI95% 283–298). Implant sizes were more than 300 cc in 38.3% of implant-IBR (176/460) and 58% of implant with LDF-IBR (29/50) (p = 0.006) (Supplementary Table 1).

LDF-IBR was performed in 1 prophylactic mastectomy (0.9%), in all 52 mastectomies after NAC-R, in 42 ILBCLR (48.8%) and in 101 primaries BC (101/496: 20.4%) including 4 among 9 mastectomies (44.4%) after previous radiotherapy for Hodgkin disease.

**Fig. 1:**
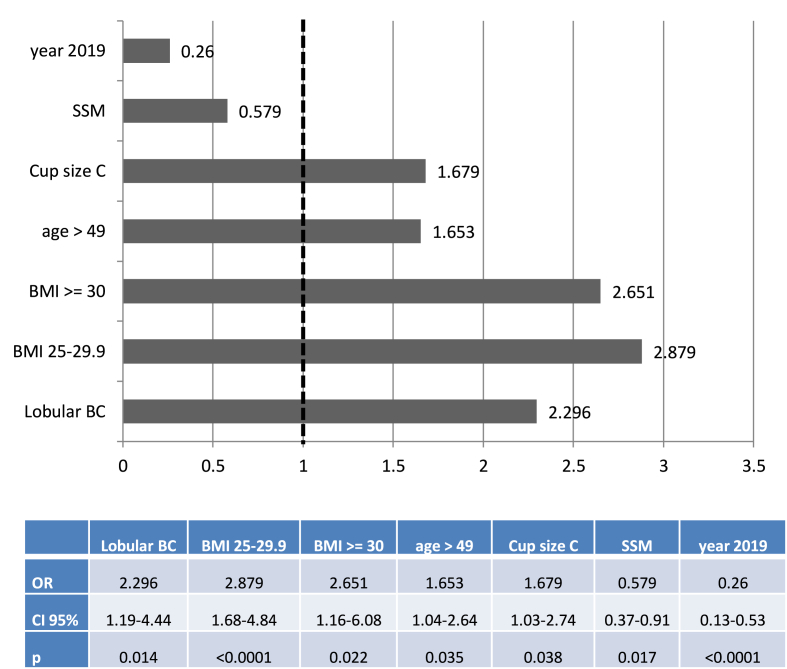
Odds ratio (OR) of regression analysis: Latissimus Dorsi Flap IBR versus Implant IBR for 579 NSM or SSM excluding prophylactic mastectomies, NAC-R and standard mastectomies. ***Legend***: OR: Odds ratio, SSM: skin sparing mastectomy, BMI: body mass index, BC: breast cancer.

*Type of reconstruction in 579 NSM or SSM, excluding 4 standard mastectomies,* 113 *prophylactic mastectomies (1 LDF-IBR) and* 52 NAC*-R (52 LDF-IBR)*: In univariate analysis, IBR with implant or with LDF ± implant, was significantly associated with indication, bilateral mastectomy, histology, years of treatment, breast cup-size, age, NSM or SSM and BMI. In multivariate analysis, IBR with LDF versus implant was associated with lobular BC, year 2019, BMI, age >49-years old, breast cup-size C and SSM (Fig. 1).

### 3.5 Duration of surgery per patients (681 patients)

Median duration of surgery was 144 min (CI95% 166–180) (Supplementary Table 1): 437 ≤ 180min (64.2%) and 244 > 180min. In univariate analysis duration >180mn was significantly associated with mastectomy weight, LDF-IBR, BMI, ALND, breast cup-size, indication and years of treatment. In multivariate analysis duration >180mn was significantly associated with breast cup-size ≥ C (OR: 1.832, CI95% 1.001–3.352, p = 0.050 for 201 cup-size C and OR: 1.980, CI95% 0.905–4.334, p = 0.087 for 108 cup-size > C) and LDF-IBR versus implant-IBR (OR: 189, CI95% 83–432, p < 0.0001) for 484 implant-IBR and 196 LDF-IBR.

### 3.6 Complications

The overall complications crude rate was 31.4% (235/748) with 74 re-operations (9.9%). Implant loss rate was 5.8% (35/599): 4.7% (26/551) for implant or expander IBR (22/459 definitive implant and 4/92 expanders) and 18.75% (9/48) for LDF with combined implant-IBR (p = 0.001). There was no significant difference between previous radiotherapy or not: 9.9% (8/81) and 5.2% (27/518), respectively (p = 0.085).

**Fig. 2:**
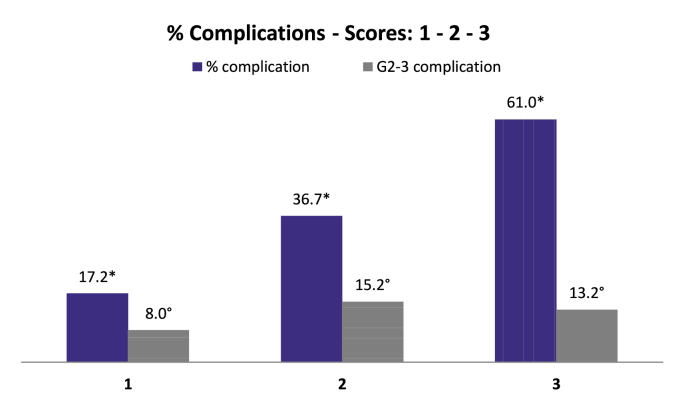
Predictive simplified score of complications. Legends: Score 1: 373 patients (50.0%), Score 2: 237 patients (31.8%), Score 3: 136 patients (18.2%). *: p < 0.0001, °: p = 0.018. Equation: tobacco (0 or 1) + type of IBR (1 or 3.265 or 3.735) + breast cup-size (1 or 1.605 or 2.147). ***Type of IBR***: Implant = 1, LDF (latissimus dorsi-flap) = 3.265, LDF + implant = 3.735. ***Breast cup-size***: A-B = 1, C = 1.605, >C = 2.147. ***Values***: Score 1: 2 to 2.60. Score 2: 3.15 to 4.85. Score 3: >4.85.

In univariate analysis complication rate was significantly associated with median age, BMI < or ≥ 25, ASA status, tobacco use, breast cup-size, NAC, years of treatment, IBR type, ALND, radiotherapy and mastectomy weight. In logistic regression, factors significantly associated with complication were: tobacco use (OR: 2.249, CI 95% 1.50–3.38, p < 0.0001), LDF-IBR (OR: 3.265, CI 95% 2.08–5.14, p < 0.0001) and LDF with Implant (OR: 3.735, CI95% 1.80–7.77, p < 0.0001) versus Implant-IBR, breast cup-size C (OR: 1.605, CI 95% 1.03–2.50, p = 0.036) and >C (OR: 2.147, CI 95% 1.24–3.73, p = 0.007) versus cup-size < C. Using these OR, predictive score of complications was between 2 and 8.14 values and was calculated with the equation reported in Fig. 2. This score was significantly associated with complication (p < 0.0001) and complication Grade 2–3 (p = 0.009). Simplified score determined 3 categories (Supplementary Table 2, Fig. 2) with a significant association with complications (AUC: 0.698, CI 95% 0.656–0.739) and complications Grade 2–3 (AUC: 0.575, CI 95% 0.512–0.638).

*Breast complications* occurred in 32.9% of mastectomies (171/748) with 89 grade 1 (52% of complications), 14 grade 2 (8.2%), 67 grade 3 (39.2%) and 1 grade 4 (septic shock with bilateral implant loss).

Type of complications were: 86 skin and or NACx suffering or necrosis (50.3%: including 21 Grade 2–3), 39 hematomas (22.8%: including 32 Grade 2–3), 26 infections (15.2%: all Grade 2–3) and 20 others complications (11.7%: including 3 Grade 2–3). Breast complications Grade 2-3-4 were significantly associated in univariate analysis only with tobacco use (p = 0.033, OR: 2.064, CI95% 1.43–2.97, p < 0.0001). Implant loss rates were significantly associated with type of complication: 15.9% (10/63) for skin or NACx suffering or necrosis, 15.2% (5/33) for hematomas, 80.0% (20/25) for infections and 0% (0/18) for others complications (p < 0.0001).

Overall breast complications rates, breast complications rates Grade ≥ 2 and skin or NACx suffering or necrosis rates for NSM according to breast incision are reported in Table 3.

**Table 3:** Breast complications rates for NSM according to breast incision.

incisions	periphery	areolar	radial	inversed T	p
% complication	25.5	26.1	24.1	72.7	<0.05
(patients number)	(51/200)	(23/88)	(13/54)	(8/11)	
G2-3 complication	13.5	15.9	1.9	18.2	0.070
(patients number)	(27/200)	(14/88)	(1/54)	(2/11)	
skin or NACx	13.5	14.8	20.4	45.4	<0.05
(patients number)	(27/200)	(13/88)	(11/54)	(5/11)	

*Legend*: G2-3: Grade 2–3, NACx: nipple areolar complex.

*LDF complications* occurred in 70 mastectomies with LDF (35.7%: 70/196): 63 grade 1 (90% of complications: dorsal seroma), 1 grade 2 (1.4%: dorsal seroma with hyperthermia) and 6 grade 3 (8.6%) with re-operation (4 hematomas, 1 infection and 1 partial LDF necrosis). There was no significant difference between robotic-LDF (R-LDF) and conventional LDF. Grade 2-3-4 complications rates were 10% for implant-IBR, 9.7% for LDF-IBR and 15.4% for LDF with implant-IBR (non-significant).

### 3.7 Post-operative hospitalization length (POHL) per patients

Median POHL was 2 days (CI95% 2.51–2.74): 2 and 4 days for implant-IBR and LDF-IBR, respectively (p < 0.0001) (Supplementary Table 1). Median POHL were 2 days (mean: 2.54, CI95% 2.43–2.65, range: 1–8) and 3 days (mean: 3.32, CI95% 2.84–3.80, range 1–14) for patients without and with complications Grade 2–3, respectively (p = 0.002).

### 3.8 Endoscopic procedures

Endoscopic NSM, 108 robotic-NSM (R-NSM) and 3 endoscopic without robotic assistance, were performed (31.4%) by 2 surgeons. R-LDF was performed for 125 IBR (63.8%: 125/196 LDF-IBR, 50 for SSM and 75 for NSM) by 3 surgeons, in 40 cases with LDF-IBR combined with implant (32%).

Durations of surgery for R-NSM, non-R-NSM, endoscopic-NSM and *for patients with LDF-IBR (Robotic or non-Robotic LDF) with SSM or non-robotic NSM* are reported in Table 4.

**Table 4:** Duration of surgery according to robotic and non-robotic procedures.

Duration of surgery	Patients number	Median	Mean	CI 95%	% LDF-IBR
NSM	304	153	183	172–194	29.6
non-robotic NSM	198	130	140	131–149	16.7
R-NSM	103	260	266	246–285	55.3
Endoscopic-NSM	3	180	181	114–248	0
non-robotic NSM implant-IBR	175	120	124	118–130	0
R-NSM implant-IBR	46	173	184	166–203	0
non-robotic LDF-IBR*	67	266	259	244–273	100
R-LDF-IBR*	68	273	285	268–301	100

*Legend*: * SSM (skin sparing mastectomy) and non-Robotic NSM, NSM: nipple sparing mastectomy, R-NSM: Robotic-NSM, IBR: immediate breast reconstruction.

*For patients with NSM and implant-IBR*, in univariate analysis, duration >130min were significantly associated with endoscopic or robotic NSM (40/49 vs 73/175: p < 0.0001), bilateral NSM (36/46 vs 77/178: p < 0.0001), mastectomy weight >300gr (58/91 vs 55/133: p = 0.001), without difference between years, breast cup-size, indication of NSM. In multivariate analysis, these factors remains significant: R-NSM (OR: 8.25, CI95% 3.55–19.2, p < 0.0001), bilateral NSM (OR: 8.05, CI95% 3.52–18.4) and mastectomy weight >300gr (OR: 2.80, CI95% 1.49–5.28, p = 0.001).

### 3.9 Pathologic results and treatment

Median mastectomy weight were 308.5gr (CI95% 340–372) with significant higher weight for SSM versus NSM and for LDF-IBR versus Implant-IBR (Supplementary Table 1).

NSM rates were significantly different according to pathologic results of mastectomies: 35.3% for DCIS, 41.8% for invasive BC and 87.6% for prophylactic mastectomies (p < 0.0001) (Table 1).

NAC was performed in 102 patients (including 52 with NAC + N-RTH) and endocrine therapy in 354 patients with primary BC (64.6%) and in 47 ILBCLR (54.0%). Previous radiotherapy was realized in 140 mastectomies (18.7%): 88 with ILBCLR or radiotherapy for Hodgkin disease and 52 patients with NAC + N-RTH. PMRT was realized in 107 mastectomies (61 with implant-IBR, 17 with expander, 22 with LDF-IBR and 7 with implant-LDF-IBR) and AC was done in 147 patients.

Median interval time between surgery and first adjuvant treatment was 44 days: 43 and 60 days for AC and PMRT, respectively. Median interval time between surgery and first adjuvant treatment were 43 and 54 days for mastectomies without and with Grade 2–3 complications, respectively (p = 0.042). According to type of complication, median interval times were not significantly different (Supplementary Table 3).

## 4 Discussion

In our study with a large number of patients, the overall complications crude rate was 31.4% with 74 re-operations (9.9%) and 35 implant losses (5.8%). Grade 2–3 breast-complication rate was 10.96%, significantly associated with tobacco use: 10% for implant-IBR, 9.7% for LDF-IBR and 15.4% for LDF-implant-IBR. There was no significant difference of complications rates between R-LDF and traditional LDF. Using our simplified score we are able to evaluate risk of complications before surgery which can help the decision and type of IBR in agreement with patient's wishes.

Even if comparison of complications rates between studies is difficult due to a great disparity of IBR types, complications recorded, indications of mastectomy and time of survey, we reported an overall complication rate similar with others studies [[32], [33], [34], [35], [36], [37], [38], [39]]. However, complications rates reported in recent studies for NSM-IBR, were lower (5.1–20%) and the average overall complication rate were 20.5% in a recent review of 3716 prophylactic-NSM [25].

We reported a 4.7% rate of implant loss for implant-IBR, mainly in relation with infectious complication, even with use of pre-operative antimicrobial therapy for patients with nasal-germs and per-operative antimicrobial-prophylaxis. This rate was lesser than rates reported by others [32,33,38]. Implant loss rate was higher for LDF with combined implant-IBR in our study. In literature, the more frequent complication was infection (0%–17.8%) with implant loss reported between 1.0% and 9.9%. Wound infection rate was 9.8% (230/2343) in Bennett et al. study [39] with a reconstructive failure rate of 7.1% (116/1637). Moreover, obesity was associated with higher risks of any complication in a recent study, in agreement with our results [40].

Major complication rate: Like others authors we observed higher breast complication rate Grade 2–3 with tobacco use (OR = 2.064): higher failure rate [41,42], higher flap necrosis rate [[43], [44], [45]] and higher infection rate [36,42,45]. Major complications rates, grade 3–4 with re-operation and/or re-hospitalization, reported in literature were comprised between 9 and 37% but with different IBR procedures, different criteria of complications recorded and different time of survey. In a multicenter prospective cohort study [39] reoperation rate was 19.3% (453/2343). In the large NMBRA-cohort with 3389 IBR, this rate was 15.8% [41]. In Srinivasa et al. study [40], obesity was significantly associated with higher risk of major complication in both implant reconstruction (OR = 1.71) and autologous reconstruction (OR = 2.72). It is interesting to note that complications Grade 2–3 had, in our study, a significant impact on interval time between surgery and adjuvant treatment with possible negative impact on prognosis. This topic was not analyzed in others studies.

We reported a high rate of NSM (47.2%) particularly during the last 2-years (56.5%). In the MROC study [37], NSM rate was 17.7% (287/1625) for implant reconstructions. Potential disadvantages of SSM and NSM include residual breast tissue under nipple-areolar-complex (NACx) or under the skin-flaps and an increased risk of mastectomy skin-flap or NACx necrosis [19]. In literature, NACx recurrence rates were very low [[46], [47], [48]] and the rate of nipple necrosis was 0.0–11% [10,[46], [47], [48], [49], [50], [51], [52], [53], [54], [55], [56], [57]]. A peri-areolar incision is considered as a risk factor of NACx necrosis and periphery incisions are considered as the best choice to preserve NACx vascularization [47,54,[57], [58], [59], [60], [61], [62]]. However, we don't observe any difference according the type of incision except for inversed T incisions with skin reducing envelop.

In our study, LDF-IBR rate was 26.2% and LDF-IBR rates were higher for high BMI, breast cup-size > C and for NSM. Autologous-IBR (LDF or others flaps pedicles or free flaps) were reported in 27.7%, 30.1%, 28% and 53.5% with LDF in 3.3%, 3.0%, 4.8% and 46.9%, respectively in studies reported by Wilkins, Bennett, Srinivasa and Dauplat [8,[38], [39], [40]].

LDF-IBR were performed in 67.1% (94/140) of our patients with previous radiotherapy. We think that LDF-reconstruction is a good choice after radiotherapy, because LDF protect and nourish skin flaps. A prospective multicenter study [23] shows that autologous reconstruction appears to yield a superior patient-reported satisfaction and lower risk of complications than implant placement among patients receiving PMRT. Sbitany et al. were specifically interested in pre-pectoral implant breast reconstruction in 175 patients compared to 236 sub-muscular reconstruction and have shown no significant differences in complication rates: 15.4% versus 19.3% [63].

Robotic surgery: Currently, only a few studies have looked at series of patients who underwent R-NSM. Sarfati et al. reported 63 prophylactic R-NSM with no mastectomy skin flap or NACx necrosis, 4.8% of infections and 1.6% implant loss [10]. For Toesca et al., with 94 R-NSM procedures, the rate of reoperation was 4.3%, flap or nipple necrosis at 1.1% and they did not highlight local recurrences [64]. Endoscopic procedure is an emerging technique that has not yet been fully validated. This allows a NSM with a unique axillary approach with endoscopic or robotic technic, which is now well determined [65] but contribution of these procedures in comparison with traditional-NSM must be confirmed by prospective studies with analysis of complication rates, aesthetics advantages and cost efficiency. All recent studies about R-NSM showed that this technic could be performed with a brief learning curve [[14], [15], [16], [17],[65], [66], [67]].

In our study we reported 236 endoscopic procedures: 111 R-NSM and 125 R-LDF. The complication rate was not higher for R-LDF than conventional-LDF, but longer duration of surgery for NSM-implant-IBR was significantly associated with the robotic procedures. A recent mono-centric study, on 91 Endoscopic-NSM and 40 R-NSM showed that R-NSM is associated with higher satisfaction but at the price of longer operation and higher medical cost [66] and two studies showed that endoscopic surgery were associated with a better esthetic outcome [[68], [69]]. Robotic-LDF appears as a safe, reproducible and contributive procedure when skin paddle is not required with any dorsal scar and less pain without significant longer procedure [15,16]. This can be explained by the enhanced surgical exposure resulting in the minimization of tissue traction and the resultant tissue trauma and skin necrosis. Clemens et al. published similar results and concluded that R-LDF harvesting is an efficient technique with low complication rate (16.7% among 12 R-LDF versus 37.5% among 64 Traditional-LDF) [70].

Robotic surgery is associated with many advantages when compared to traditional surgery such as a smaller incision, better surgical exposure, decreased tissue trauma and an enhanced viability of the LDF. Acquiring a good experience in robotic surgery (pelvic or breast) is considered crucial: this can be accomplished while assisting more experienced surgeons especially with a dual robot console.

Radiotherapy: In a recent review, Ho et al. [71] wondered if IBR and PMRT combination were possible while minimizing the frequency of complications without compromising oncological or cosmetic outcomes. It seemed like IBR and PMRT were compatible. Otherwise autologous reconstructions tolerate radiotherapy better compared with implants. However, implants remain the predominant type of reconstruction because it preserve the option of delayed autologous reconstruction [71]. In our study PMRT was realized for 107 patients with implant-IBR (72.9%). Reverberi et al. have shown that the type of reconstruction does not influence late toxicity rate: 25.3% among 91 IBR with PMRT [72]. Regarding oncological safety of IBR with PMRT, Bjohle et al. [73] in a matched cohort study with implant-IBR patients (n = 128) compared to patients without implant (n = 252) observed no difference in survival and recurrence [73].

Several limits of our study can be underlined: post-operative complications were recorded only during 90-days and we can't evaluate patient's satisfaction and quality of life.

## 5 Conclusion

In conclusion, IBR were performed in 37.7% of mastectomies and after RTH in 18.9%, with NSM in 47.2% and more NSM during the 2 last years. LDF-IBR was performed in 26.2% of all mastectomies with IBR. More LDF-IBR for high BMI and high breast cup-size were performed for primary BC and LDF-IBR were frequently performed for patients with NAC-R and local recurrence.

Mastectomy with IBR for local recurrence seems to be a safe technique with an acceptable complication rate, which is increased by tobacco use. This technique can be proposed after a strict selection of patient's characteristics and informed the patient of the risk of increasing the interval time for adjuvant treatments in the event of complications. Predictive score to evaluate the complication rate could be used to informed patients to help the decision with patient wishes. Otherwise, robotic surgery is associated with many advantages but needs complementary evaluation by a prospective study.

## Guarantor

Prof. Gilles Houvenaeghel.

## Funding

This research did not receive any specific grant from funding agencies in the public, commercial, or not-for-profit sectors.

## Declaration of competing interest

The authors declare that there are no conflicts of interest regarding this study.

## Data statement

The database used and/or analyzed during the current study are not publicly available, but can be available from the corresponding author on reasonable request.

## Research registration Unique Identifying number (UIN)

Name of the registry: Database of Data Collection in Senological Surgery.

Unique Identifying number or registration ID: ClinicalTrials.gov Identifier: NCT03461172.

Hyperlink to your specific registration (must be publicly accessible and will be checked): https://clinicaltrials.gov/ct2/show/NCT03461172.

## References

[1] Krag D.N., Anderson S.J., Julian T.B., Brown A.M., Harlow S.P., Costantino J.P. (2010). Sentinel-lymph-node resection compared with conventional axillary-lymph-node dissection in clinically node-negative patients with breast cancer: overall survival findings from the NSABP B-32 randomised phase 3 trial. Lancet Oncol..

[2] Houvenaeghel G., Cohen M., Raro P., De Troyer J., de Lara C.T. (2018). Overview of the pathological results and treatment characteristics in the first 1000 patients randomized in the SERC trial: axillary dissection versus no axillary dissection in patients with involved sentinel node. BMC Canc..

[3] Houvenaeghel G., Lambaudie E., Cohen M., Classe J.-M., Reyal F., Garbay J.-R. (2017). Therapeutic escalation - de-escalation: data from 15.508 early breast cancer treated with upfront surgery and sentinel lymph node biopsy (SLNB). Breast. août.

[4] Houvenaeghel G., Lambaudie E., Bannier M., Rua S., Barrou J., Heinemann M. (2019). Positive or close margins: reoperation rate and second conservative resection or total mastectomy?. Canc. Manag. Res..

[5] Nègre G., Balcaen T., Dast S., Sinna R., Chazard E. (2020). Breast reconstruction in France, observational study of 140,904 cases of mastectomy for breast cancer. Ann. Chir. Plast Esthet..

[6] Recommendations professionnelles cancer du Sein in situ [internet]. https://studylibfr.com/doc/3647130/recommandations-professionnelles-cancer-du-sein-in-situ.

[7] ALD n° 30 - cancer du sein [Internet]. https://www.has-sante.fr/jcms/c_927251/fr/ald-n-30-cancer-du-sein.

[8] Dauplat J., Kwiatkowski F., Rouanet P., Delay E., Clough K., Verhaeghe J.L. (2017). Quality of life after mastectomy with or without immediate breast reconstruction. Br. J. Surg..

[9] Offodile A.C., Lee C.N.-H. (2018). Future directions for breast reconstruction on the 20th anniversary of the women's health and cancer rights act. JAMA Surg.

[10] Sarfati B., Struk S., Leymarie N., Honart J.-F., Alkhashnam H., Tran de Fremicourt K. (2018). Robotic prophylactic nipple-sparing mastectomy with immediate prosthetic breast reconstruction: a prospective study. Ann. Surg. Oncol..

[11] Struk S., Qassemyar Q., Leymarie N., Honart J.-F., Alkhashnam H., De Fremicourt K. (2018). The ongoing emergence of robotics in plastic and reconstructive surgery. Ann. Chir. Plast. Esthet..

[12] Toesca A., Peradze N., Galimberti V., Manconi A., Intra M., Gentilini O. (2017). Robotic nipple-sparing mastectomy and immediate breast reconstruction with implant: first report of surgical technique. Ann. Surg..

[13] Lai H.-W., Chen S.-T., Lin S.-L., Chen C.-J., Lin Y.-L., Pai S.-H. (2019). Robotic nipple-sparing mastectomy and immediate breast reconstruction with gel implant: technique, preliminary results and patient-reported cosmetic outcome. Ann. Surg. Oncol..

[14] Lai H.-W., Wang C.-C., Lai Y.-C., Chen C.-J., Lin S.-L., Chen S.-T. (2019). The learning curve of robotic nipple sparing mastectomy for breast cancer: an analysis of consecutive 39 procedures with cumulative sum plot. Eur. J. Surg. Oncol..

[15] Houvenaeghel G., Bannier M., Rua S., Barrou J., Heinemann M., Lambaudie E. (2019). Skin sparing mastectomy and robotic latissimus dorsi-flap reconstruction through a single incision. World J. Surg. Oncol..

[16] Houvenaeghel G., Bannier M., Rua S., Barrou J., Heinemann M., Knight S. (2019). Robotic breast and reconstructive surgery: 100 procedures in 2-years for 80 patients. Surg. Oncol..

[17] Houvenaeghel G., Bannier M., Rua S., Barrou J., Heinemann M., Van Troy A. (2019). Breast cancer robotic nipple sparing mastectomy: evaluation of several surgical procedures and learning curve. World J. Surg. Oncol..

[18] Hartmann L.C., Schaid D.J., Woods J.E., Crotty T.P., Myers J.L., Arnold P.G. (1999). Efficacy of bilateral prophylactic mastectomy in women with a family history of breast cancer. N Engl. J. Med..

[19] Meijers-Heijboer H., van Geel B., van Putten W.L., Henzen-Logmans S.C., Seynaeve C., Menke-Pluymers M.B. (2001). Breast cancer after prophylactic bilateral mastectomy in women with a BRCA1 or BRCA2 mutation. N Engl. J. Med..

[20] Contant C.M.E., Menke-Pluijmers M.B.E., Seynaeve C., Meijers-Heijboer E.J., Klijn J.G.M., Verhoog L.C. (2002). Clinical experience of prophylactic mastectomy followed by immediate breast reconstruction in women at hereditary risk of breast cancer (HB(O)C) or a proven BRCA1 and BRCA2 germ-line mutation. Eur. J. Surg. Oncol..

[21] Wei C.H., Scott A.M., Price A.N., Miller H.C., Klassen A.F., Jhanwar S.M. (2016). Psychosocial and sexual well-being following nipple-sparing mastectomy and reconstruction. Breast J..

[22] Mota B.S., Riera R., Ricci M.D., Barrett J., de Castria T.B., Atallah Á.N. (2016). Nipple- and areola-sparing mastectomy for the treatment of breast cancer. Cochrane Database Syst. Rev..

[23] Jagsi R., Li Y., Morrow M., Janz N., Alderman A., Graff J. (2015). Patient-reported quality of life and satisfaction with cosmetic outcomes after breast conservation and mastectomy with and without reconstruction: results of a survey of breast cancer survivors. Ann. Surg..

[24] Li M., Chen K., Liu F., Su F., Li S., Zhu L. (2017). Nipple sparing mastectomy in breast cancer patients and long-term survival outcomes: an analysis of the SEER database. PloS One.

[25] Muller T., Baratte A., Bruant-Rodier C., Bodin F., Mathelin C. (2018). Oncological safety of nipple-sparing prophylactic mastectomy: a review of the literature on 3716 cases. Ann. Chir. Plast. Esthet..

[26] Wu Z.-Y., Kim H.-J., Lee J.-W., Chung I.-Y., Kim J.-S., Lee S.-B. (1 nov 2019). Breast cancer recurrence in the nipple-areola complex after nipple-sparing mastectomy with immediate breast reconstruction for invasive breast cancer. JAMA Surg.

[27] Jakub J.W., Peled A.W., Gray R.J., Greenup R.A., Kiluk J.V., Sacchini V. (2018). Oncologic safety of prophylactic nipple-sparing mastectomy in a population with *BRCA* mutations: a multi-institutional study. JAMA Surg..

[28] De Vita R., Zoccali G., Buccheri E.M., Costantini M., Botti C., Pozzi M. (2017). Outcome evaluation after 2023 nipple-sparing mastectomies: our experience. Plast. Reconstr. Surg..

[29] Qureshi A.A., Odom E.B., Parikh R.P., Myckatyn T.M., Tenenbaum M.M. (2017). Patient-reported outcomes of aesthetics and satisfaction in immediate breast reconstruction after nipple-sparing mastectomy with implants and fat grafting. Aesthet Surg J.

[30] Agha R., Abdall-Razak A., Crossley E., Dowlut N., Iosifidis C., Mathew G., for the STROCSS Group (2019). The STROCSS 2019 guideline: strengthening the reporting of cohort studies in surgery. Int. J. Surg..

[31] Dindo D., Demartines N., Clavien P.-A. (2004). Classification of surgical complications: a new proposal with evaluation in a cohort of 6336 patients and results of a survey. Ann. Surg..

[32] Pinsolle V., Grinfeder C., Mathoulin-Pelissier S., Faucher A. (2006). Complications analysis of 266 immediate breast reconstructions. J. Plast. Reconstr. Aesthetic Surg..

[33] Alderman A.K., Wilkins E.G., Kim H.M., Lowery J.C. (2002). Complications in postmastectomy breast reconstruction: two-year results of the Michigan Breast Reconstruction Outcome Study. Plast. Reconstr. Surg..

[34] Berry T., Brooks S., Sydow N., Djohan R., Nutter B., Lyons J. (2010). Complication rates of radiation on tissue expander and autologous tissue breast reconstruction. Ann. Surg Oncol..

[35] Contant C.M., van Geel A.N., van der Holt B., Griep C., Tjong Joe Wai R., Wiggers T. (2000). Morbidity of immediate breast reconstruction (IBR) after mastectomy by a subpectorally placed silicone prosthesis: the adverse effect of radiotherapy. Eur. J. Surg. Oncol..

[36] Ducic I., Spear S.L., Cuoco F., Hannan C. (2005). Safety and risk factors for breast reconstruction with pedicled transverse rectus abdominis musculocutaneous flaps: a 10-year analysis. Ann. Plast. Surg..

[37] Headon H.L., Kasem A., Mokbel K. (2016). The oncological safety of nipple-sparing mastectomy: a systematic review of the literature with a pooled analysis of 12,358 procedures. Arch. Plast. Surg..

[38] Wilkins E.G., Hamill J.B., Kim H.M., Kim J.Y., Greco R.J., Qi J. (2018). Complications in postmastectomy breast reconstruction: one-year outcomes of the mastectomy reconstruction outcomes consortium (MROC) study. Ann. Surg..

[39] Bennett K.G., Qi J., Kim H.M., Hamill J.B., Pusic A.L., Wilkins E.G. (2018). Comparison of 2-year complication rates among common techniques for postmastectomy breast reconstruction. JAMA Surg.

[40] Srinivasa D.R., Clemens M.W., Qi J., Hamill J.B., Kim H.M., Pusic A.L. (2020). Obesity and breast reconstruction: complications and patient-reported outcomes in a multicenter, prospective study. Plast. Reconstr. Surg..

[41] reportNational Mastectomy and Breast Reconstruction Audit – Third Annual Report 2010.

[42] McCarthy C.M., Mehrara B.J., Riedel E., Davidge K., Hinson A., Disa J.J. (2008). Predicting complications following expander/implant breast reconstruction: an outcomes analysis based on preoperative clinical risk. Plast. Reconstr. Surg..

[43] Petersen A., Eftekhari A.L.B., Damsgaard T.E. (2012). Immediate breast reconstruction: a retrospective study with emphasis on complications and risk factors. J. Plast. Surg. Hand. Surg..

[44] Albino F.P., Koltz P.F., Ling M.N., Langstein H.N. (2010). Irradiated autologous breast reconstructions: effects of patient factors and treatment variables. Plast. Reconstr. Surg..

[45] Chang D.W., Reece G.P., Wang B., Robb G.L., Miller M.J., Evans G.R. (2000). Effect of smoking on complications in patients undergoing free TRAM flap breast reconstruction. Plast. Reconstr. Surg..

[46] Petit J.Y., Veronesi U., Orecchia R., Rey P., Martella S., Didier F. (2009). Nipple sparing mastectomy with nipple areola intraoperative radiotherapy: one thousand and one cases of a five years experience at the European institute of oncology of Milan (EIO). Breast Canc. Res. Treat..

[47] Féron J.-G., Leduey A., Mallon P., Couturaud B., Fourchotte V., Guillot E. (2014). [The role of nipple-sparing mastectomy in breast cancer: a comprehensive review of the literature]. Ann. Chir. Plast. Esthet..

[48] De La Cruz L., Moody A.M., Tappy E.E., Blankenship S.A., Hecht E.M. (2015). Overall survival, disease-free survival, local recurrence, and nipple-areolar recurrence in the setting of nipple-sparing mastectomy: a meta-analysis and systematic review. Ann. Surg Oncol..

[49] Vega S., Smartt J.M., Jiang S., Selber J.C., Brooks C.J.M., Herrera H.R. (2008). 500 Consecutive patients with free TRAM flap breast reconstruction: a single surgeon's experience. Plast. Reconstr. Surg..

[50] Gould D.J., Hunt K.K., Liu J., Kuerer H.M., Crosby M.A., Babiera G. (2013). Impact of surgical techniques, biomaterials, and patient variables on rate of nipple necrosis after nipple-sparing mastectomy. Plast. Reconstr. Surg..

[51] de Alcantara Filho P., Capko D., Barry J.M., Morrow M., Pusic A., Sacchini V.S. (2011). Nipple-sparing mastectomy for breast cancer and risk-reducing surgery: the Memorial Sloan-Kettering Cancer Center experience. Ann. Surg Oncol..

[52] Warren Peled A., Foster R.D., Stover A.C., Itakura K., Ewing C.A., Alvarado M. (oct 2012). Outcomes after total skin-sparing mastectomy and immediate reconstruction in 657 breasts. Ann. Surg Oncol..

[53] Wagner J.L., Fearmonti R., Hunt K.K., Hwang R.F., Meric-Bernstam F., Kuerer H.M. (2012). Prospective evaluation of the nipple-areola complex sparing mastectomy for risk reduction and for early-stage breast cancer. Ann. Surg. Oncol..

[54] Colwell A.S., Tessler O., Lin A.M., Liao E., Winograd J., Cetrulo C.L. (2014). Breast reconstruction following nipple-sparing mastectomy: predictors of complications, reconstruction outcomes, and 5-year trends. Plast. Reconstr. Surg..

[55] Klein J., Kong I., Paszat L., Nofech-Mozes S., Hanna W., Thiruchelvam D. (2015). Close or positive resection margins are not associated with an increased risk of chest wall recurrence in women with DCIS treated by mastectomy: a population-based analysis. SpringerPlus.

[56] Lago V., Maisto V., Gimenez-Climent J., Vila J., Vazquez C., Estevan R. (2018). Nipple-sparing mastectomy as treatment for patients with ductal carcinoma in situ: a 10-year follow-up study. Breast J..

[57] Lam G.-T., Feron J.-G., Mallon P., Roulot A., Couturaud B. (2018). The inframammary skin-sparing mastectomy technique. Ann. Chir. Plast. Esthet..

[58] Endara M., Chen D., Verma K., Nahabedian M.Y., Spear S.L. (2013). Breast reconstruction following nipple-sparing mastectomy: a systematic review of the literature with pooled analysis. Plast. Reconstr. Surg..

[59] Salgarello M., Visconti G., Barone-Adesi L. (2010). Nipple-sparing mastectomy with immediate implant reconstruction: cosmetic outcomes and technical refinements. Plast. Reconstr. Surg..

[60] Weber W.P., Haug M., Kurzeder C., Bjelic-Radisic V., Koller R., Reitsamer R. (2018). Oncoplastic Breast Consortium consensus conference on nipple-sparing mastectomy. Breast Canc. Res. Treat..

[61] Donovan C.A., Harit A.P., Chung A., Bao J., Giuliano A.E., Amersi F. (2016). Oncological and surgical outcomes after nipple-sparing mastectomy: do incisions matter?. Ann. Surg Oncol..

[62] Ashikari A.Y., Kelemen P.R., Tastan B., Salzberg C.A., Ashikari R.H. (2018). Nipple sparing mastectomy techniques: a literature review and an inframammary technique. Gland. Surg..

[63] Sbitany H., Gomez-Sanchez C., Piper M., Lentz R. (2019). Prepectoral breast reconstruction in the setting of postmastectomy radiation therapy: an assessment of clinical outcomes and benefits. Plast. Reconstr. Surg..

[64] Toesca A., Invento A., Massari G., Girardi A., Peradze N., Lissidini G. (2019). Update on the feasibility and progress on robotic breast surgery. Ann. Surg Oncol..

[65] Lai H.-W., Toesca A., Sarfati B., Park H.S., Houvenaeghel G., Selber J.C. (2020). Consensus statement on robotic mastectomy—expert panel from international endoscopic and robotic breast surgery symposium (IERBS) 2019. ann. Surg..

[66] Lai H.-W., Chen S.-T., Tai C.-M., Lin S.-L., Lin Y.-J., Huang R.-H. (2020). Robotic- versus endoscopic-assisted nipple-sparing mastectomy with immediate prosthesis breast reconstruction in the management of breast cancer: a case-control comparison study with analysis of clinical outcomes, learning curve, patient-reported aesthetic results, and medical cost. Ann. Surg. Oncol..

[67] Ahn S.J., Song S.Y., Park H.S., Park S.H., Lew D.H., Roh T.S. (2019). Early experiences with robot-assisted prosthetic breast reconstruction. Arch. Plast. Surg..

[68] Tukenmez M., Ozden B.C., Agcaoglu O., Kecer M., Ozmen V., Muslumanoglu M. (2014). Videoendoscopic single-port nipple-sparing mastectomy and immediate reconstruction. J. Laparoendosc. Adv. Surg. Tech. A.

[69] Kitamura K., Ishida M., Inoue H., Kinoshita J., Hashizume M., Sugimachi K. (2002). Early results of an endoscope-assisted subcutaneous mastectomy and reconstruction for breast cancer. Surgery.

[70] Clemens M.W., Kronowitz S., Selber J.C. (2014). Robotic-assisted latissimus dorsi harvest in delayed-immediate breast reconstruction. Semin. Plast. Surg..

[71] Ho A.Y., Hu Z.I., Mehrara B.J., Wilkins E.G. (2017). Radiotherapy in the setting of breast reconstruction: types, techniques, and timing. Lancet Oncol..

[72] Reverberi C., Marinelli L., Campanella B., Scalabrino G., Nicosia L., Anzellini D. (2020). Post-mastectomy immediate breast reconstruction and adjuvant radiotherapy: long term results of a mono institutional experience. Radiol. Med..

[73] Bjöhle J., Onjukka E., Rintelä N., Eloranta S., Wickman M., Sandelin K. (2019). Post-mastectomy radiation therapy with or without implant-based reconstruction is safe in terms of clinical target volume coverage and survival – a matched cohort study. Radiother. Oncol..

