# Supplementary material for: Mastectomy with immediate breast reconstruction: Results of a mono-centric 4-years cohort
Source: Ann Med Surg (Lond). 2020 Dec 31;61:172–9. doi: 10.1016/j.amsu.2020.12.033 (PMC7787913; doi:10.1016/j.amsu.2020.12.033)
Supplement: Multimedia component 1 [file mmc1.docx]

**Supplementary table 1 :** Median and mean values of age, BMI, post-operative hospitalization length, duration of surgery, mastectomy weight and implant size.

|  |  | Median | Mean | Test-t |
| --- | --- | --- | --- | --- |
|  |  | **All patients** |  | p |
| age | All | 50 | 51.1 |  |
|  | NSM | 48 | 49.9 | 0.032 |
|  | SSM | 51.0 | 52.0 |  |
|  | IBR with implant | 49.0 | 50.1 | 0.002 |
|  | IBR with LDF | 52.0 | 53.4 |  |
| BMI | All | 22.0 | 22.9 |  |
|  | NSM | 21.1 | 22.1 | <0.0001 |
|  | SSM | 22.6 | 23.5 |  |
|  | IBR with implant | 21.3 | 22.2 | <0.0001 |
|  | IBR with LDF | 23.5 | 24.6 |  |
| POHL | All | 2.0 | 2.63 |  |
|  | NSM | 2.0 | 2.70 | 0.261 |
|  | SSM | 2.0 | 2.57 |  |
|  | IBR with implant | 2.0 | 2.11 | <0.0001 |
|  | IBR with LDF | 4.0 | 3.92 |  |
| Duration surgery | All | 144 | 173 |  |
|  | NSM | 153 | 183 | 0.008 |
|  | SSM | 136 | 164 |  |
|  | IBR with implant | 120 | 126 | <0.0001 |
|  | IBR with LDF | 280 | 288 |  |
|  |  | **All mastectomies** | |  |
| Mastectomy weight | All | 309 | 356 |  |
|  | NSM | 277 | 313 | <0.0001 |
|  | SSM | 335 | 392 |  |
|  | IBR with implant | 287 | 329 | <0.0001 |
|  | IBR with LDF | 386 | 435 |  |
| Implant size | All | 290 | 291 |  |
|  | NSM | 290 | 293 | 0.650 |
|  | SSM | 290 | 289 |  |
|  | IBR with implant | 280 | 287 | 0.006 |
|  | IBR with LDF | 340 | 326 |  |

***Legend***: NSM : nipple sparing mastectomy, SSM: skin sparing mastectomy, IBR: immediate breast reconstruction, LDF : latissimus dorsi-flap, BMI: body mass index, POHL: post-operative hospitalization length. Age: years, duration of surgery: minutes, implant size: centimeters^3^, mastectomy weight: grams, POHL: days.

**Supplementary table 2 :** Predictive score of complications.

| Simplified score predictive of complication | | |  |  |  |
| --- | --- | --- | --- | --- | --- |
| score value | 2 to 2.60 | 3.15 to 4.85 | > 4.85 | AUC | p |
| complication rate | 17.2 | 36.7 | 61.0 | 0.698 | <0.0001 |
| complication G2-3 rate | 8.0 | 15.2 | 13.2 | 0.575 | 0.018 |
| patients number (%) | 373 (50.0%) | 237 (31.8%) | 136 (18.2%) |  |  |

**Supplementary table 3** : Interval time between surgery and first adjuvant treatment

|  | Days |  |  |  | <=60 | > 60 days | >60 |
| --- | --- | --- | --- | --- | --- | --- | --- |
|  | Median | Mean | CI95% | range | nb | nb | % |
| All Adjuvant treatment | 44 | 49 | 45.6-52.7 | 11-167 | 122 | 30 | 19.7 |
| AC | 43 | 46.5 | 43-50 | 11-167 | 111 | 20 | 15.3** |
| PMRT | 60 | 65.4 | 55.6-75.3 | 32-107 | 11 | 10 | 47.6** |
| Without complication Grade 2-3 | 43°° | 47.8 | 44-51 | 11-167 | 109 | 23 | 17.4° |
| With Grade 2-3 complication | 54°° | 58.8 | 46-71 | 28-122 | 13 | 7 | 65.0° |
| Skin or NAC suffering/necrosis | 54 | 57 | 41-73 | 21-135 | 11 | 4 | 26.7* |
| Hematomas | 46.5 | 49 | 26-71 | 11-100 | 6 | 3 | 33.3* |
| Infection | 62 | 77 | 33-121 | 43-122 | 2 | 3 | 60.0* |
| Others complications | 41 | 41 | 16-66 | 31-51 | 3 | 0 | 0.0* |
| without complication | 43 | 47 | 43-51 | 11-167 | 91 | 17 | 15.7 |

***Legends***: ** 0.002, °° 0.042 Test-t, ° 0.067, * 0.327, AC: adjuvant chemotherapy, PMRT: post mastectomy radiotherapy, nb: number.
